# New-Onset Atrial Fibrillation in St-Segment Elevation Myocardial Infarction: Predictors and Impact on Therapy And Mortality

**DOI:** 10.5935/abc.20190190

**Published:** 2019-11

**Authors:** Kisa Hyde Congo, Adriana Belo, João Carvalho, David Neves, Rui Guerreiro, João António Pais, Diogo Brás, Mafalda Carrington, Bruno Piçarra, Ana Rita Santos, José Aguiar

**Affiliations:** 1 Hospital Espírito Santo de Évora, Évora - Portugal; 2 Centro Nacional Coleção de Dados em Cardiologia, Coimbra - Portugal

**Keywords:** Atrial Fibrillation, ST Elevation Myocardial Infarction/complications, Hospitalization, Mortality, Antihypertensive, Anticoagulants

## Abstract

**Backgrund:**

New-onset atrial fibrillation complicating acute myocardial infarction represents an important challenge, with prognostic significance.

**Objective:**

To study the incidence, impact on therapy and mortality, and to identify predictors of development of new-onset atrial fibrillation during hospital stay for ST-segment elevation myocardial infarction.

**Methods:**

We studied all patients with ST-elevation myocardial infarction included consecutively, between 2010 and 2017, in a Portuguese national registry and compared two groups: 1 - no atrial fibrillation and 2 - new-onset atrial fibrillation. We adjusted a logistic regression model data analysis to assess the impact of new-onset atrial fibrillation on in-hospital mortality and to identify independent predictors of its development. A p value < 0.05 was considered significant.

**Results:**

We studied 6325 patients, and new-onset atrial fibrillation was found in 365 (5.8%). Reperfusion was successfully accomplished in both groups with no difference regarding type of reperfusion. In group 2, therapy with beta-blockers and angiotensin-conversion enzyme (ACE) inhibitors/angiotensin receptor blockers (ARBs) was less frequent, 20.6% received anticoagulation at discharge and 16.1% were on triple therapy. New-onset atrial fibrillation was associated with more in-hospital complications and mortality. However, it was not found as an independent predictor of in-hospital mortality. We identified age, prior stroke, inferior myocardial infarction and complete atrioventricular block as independent predictors of new-onset atrial fibrillation.

**Conclusion:**

New-onset atrial fibrillation remains a frequent complication of myocardial infarction and is associated with higher rate of complications and in-hospital mortality. Age, prior stroke, inferior myocardial infarction and complete atrioventricular block were independent predictors of new onset atrial fibrillation. Only 36.7% of the patients received anticoagulation at discharge.

## Introduction

Atrial fibrillation (AF) is the most commonly encountered arrhythmia and often complicates acute myocardial infarction (AMI) with new-onset atrial fibrillation of 6-21%.^1^ In this clinical setting, the occurrence of AF is significant, since rapid and irregular ventricular rates can further impair coronary circulation. The etiology of AF in this context, as in critical illnesses, is most likely multifactorial and includes ischemia, reduced atrial perfusion, glycolytic anaerobic pathways, inflammation, neurohumoral factors, autonomic regulation abnormalities, high left ventricular end-diastolic pressure and elevated atrial pressure.^2-4^ The bulk of evidence demonstrates that AF in patients hospitalized for AMI represents an important challenge, concerning the role of antiarrhythmic drugs and antithrombotic treatment.^1,2^ The development of AF is linked to poorer prognosis and adverse impact on in-hospital and long-term mortality.^5,6^ This seems to also apply for transient AF, which has reversed back to sinus rhythm at the time of discharge.

With advancements in reperfusion strategies and contemporary treatment with angiotensin-conversion enzyme (ACE) inhibitors, statins and antiplatelet therapy, it could be anticipated that there would be a change in incidence and prognostic impact of AF in the setting of AMI.^2^

The purpose of this study was to determine the incidence of AF in patients hospitalized for ST-segment elevation myocardial infarction (STEMI), identify predictors of AF development and analyze its impact on therapy and in-hospital mortality, in the setting of AMI managed according to the most recent guideline strategies.

## Methods

We performed a multicenter retrospective cohort study and identified all hospital admissions from October 2010 to August 2017 with primary discharge diagnosis of ST-segment elevation myocardial infarction included consecutively in a national multicenter Portuguese registry on Acute Coronary Syndromes (ProACS). This Portuguese registry includes 25 cardiology departments and has received the approval and authorization from the National Committee of Data Protection (authorization number 3140/2010) and is registered at ClinicalTrials.gov with identification number NCT 01642329.

We used the third universal definition of STEMI as a new ST-segment elevation at the J point ≥ 0.1 mV in two contiguous leads or new left bundle branch block, for over 30 minutes, in a clinical setting consistent with acute myocardial ischemia.

New-onset AF was defined as first occurrence of AF at the time of the myocardial infarction (MI) or any time after MI onset, in the absence of prior diagnosis.

We divided the patients into 2 groups: Group 1 - Patients who did not develop AF during in-hospital stay for STEMI and Group 2 - Patients with new-onset AF during in-hospital stay for STEMI. We excluded patients with previous diagnosis of AF or previous anticoagulation therapy.

In each patient, baseline clinical characteristics, including demographics and patient history (cardiovascular and non-cardiovascular comorbidities) were collected. Data relating to coronary angiography, percutaneous coronary intervention (namely, number of affected vessels and number of treated vessels) left ventricular function (assessed either by echocardiography, magnetic resonance, computed tomography, angiography or radionuclide imaging) and in-hospital and post-discharge medical therapy was also analyzed. The outcome variables studied were in-hospital mortality and complications, namely: heart failure, cardiogenic shock, stroke, major bleeding, atrioventricular (AV) block, ventricular tachycardia, cardiac arrest, mechanical complications of AMI and need for invasive mechanical ventilation, Swan-Ganz catheter, transvenous cardiac pacing, intra-aortic balloon pump or ventricular assist device.

We compared the defined outcomes in both groups and performed multivariate data analysis to assess independent predictors for new-onset AF and the impact of new-onset AF on in-hospital mortality.

The study protocol is in accordance with the Declaration of Helsinki.

### Data analysis

Categorical variables were characterized by absolute and relative frequencies, and numerical variables by means and standard deviations for symmetric distributions; for asymmetric distributions, medians and interquartile intervals were used. Normality was assessed by the Kolmogorov-Smirnov test.

Comparisons between both groups regarding categorical variables were conducted using the chi-square Test or Fisher’s exact Test. For the continuous variables, unpaired t-tests were used to compare the means whenever possible, otherwise, the Mann-Whitney *U* test was used to compare the medians.

Predictors were determined by adjusting the logistic regression model. Variables were selected to be included in the model using the stepwise (forward) method, together with the likelihood-ratio test. For each variable included in the regression model, adjusted odds ratio and 95% confidence interval (CI95%) were also estimated. The quality of the adjustment of the models was assessed by determining the area under the Receiver Operating Characteristic curve (AUC) and its sensitivity and specificity.

A Kaplan-Meier estimate was obtained for time to death. Comparison of two survival functions was conducted by log-rank test.

Statistical analyses were conducted using SPSS 19.0^®^ at a 5% significance level for hypothesis-testing.

## Results

**Figure 1:**
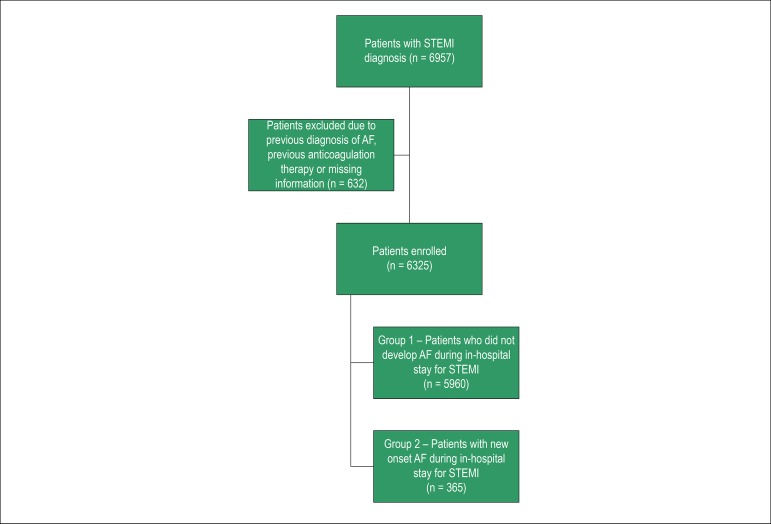
Flowchart with patients included and excluded.

A total of 6957 patients with primary diagnosis of ST-segment elevation myocardial infarction were identified. We excluded 632 patients due to previous diagnosis of AF, previous anticoagulation therapy or missing information. A total of 6325 patients were enrolled (Figure 1). New-onset AF was found in 365 patients (5.8%). Overall baseline characteristics and of both groups are summarized in Table 1.

**Table 1:** Baseline characteristics

N	All	Group 1 - No AF	Group 2 - New onset AF	p	OR (CI 95%)
	6325	5960 (94.2%)	365 (5.8%)		
Female (%)	24.2	23.6	34.2	< 0.001	1.69 (1.35-2.12)
Age (years) ≥ 75 (%)	63 ± 13	62 ± 13	71 ± 13	< 0.001	3.24 (2.61-4.01)
	21.6	20.1	44.9	< 0.001	
BMI >30 (%)	20.1	20.2	18.3	0.413	0.89 (0.67-1.18)
Arterial hypertension (%)	59.6	59.0	69.1	< 0.001	1.55 (1.23-1.95)
Diabetes mellitus (%)	23.7	23.4	28.8	0.021	1.32 (1.04-1.67)
Dyslipidemia (%)	51.1	51.4	47.7	0.186	0.86 (0.69-1.07)
Smoking habits (%)	38.6	39.3	28.6	< 0.001	0.55 (0.43-0.69)
Family history of coronary disease (%)	8.5	8.8	2.4	< 0.001	0.25 (0.12-0.53)
Prior MI (%)	10.1	10.1	9.9	0.881	0.97 (0.68-1.39)
Prior PCI (%)	8.5	8.7	5.8	0.051	0.64 (0.41-1.00)
Prior CABG (%)	1.0	0.9	2.5	0.011	2.71 (1.33-5.52)
Valvular heart disease (%)	0.9	0.7	3.4	< 0.001	4.61 (2.41-8.81)
History of heart failure (%)	1.4	1.3	3.6	< 0.001	2.81 (1.55-5.11)
Previous stroke (%)	5.4	5.0	10.4	< 0.001	2.19 (1.53-3.12)
Peripheral artery disease (%)	2.6	2.5	5.0	0.004	2.06(1.25-3.40)
Pacemaker/ ICD (%)	0.4	0.4	0.5	0.647	1.48 (0.35-6.32)
Chronic renal disease (%)	2.9	2.8	4.1	0.132	1.51 (0.88-2.59)
CPOD (%)	3.4	3.2	6.9	< 0.001	2.23 (1.45-3.43)
Dementia (%)	1.9	1.7	5.3	< 0.001	3.18 (1.92-5.27)

Values are mean±SD, median (P25; P75) or n (%). AF: atrial fibrillation; BMI: body mass index; MI: myocardial infarction; PCI: percutaneous coronary intervention; CABG: coronary artery bypass graft; ICD: implantable cardio-defibrillator; COPD: chronic obstructive pulmonary disease.

Regarding patient demographics and comorbidities, patients who developed new-onset AF were older, with higher prevalence of female sex, higher prevalence of hypertension, diabetes mellitus, prior coronary artery bypass grafting (CABG), valvular heart disease, prior heart failure (HF), prior stroke, peripheral arterial disease, chronic obstructive pulmonary disease (COPD) and dementia. There was lower prevalence of smoking habits and family history of coronary disease in group 2. No difference between both groups was found regarding the other characteristics analyzed.

Clinical data at admission is shown in Table 2. Patients in group 2 had higher heart rates and lower systolic blood pressure at admission. Patients in group 2 presented heart failure more frequently.

**Table 2:** Clinical data at admission

	All	No AF	New onset AF	p
**AMI (%)**				
- Anterior	48.4	48.4	49.0	0.806
- Inferior	50.6	50.6	49.6	0.704
- LBBB	1.0	1.0	1.4	0.424
Heart rate (bpm) >100 bpm (%)	77 ± 19	77 ± 18	79 ± 24	0.066
	11.7	11.3	18.7	< 0.001
Systolic arterial pressure (mmHg) < 100 mmHg (%)	135 ± 30	135 ± 30	126 ± 28	< 0.001
	12.3	12.0	18.1	0.001
Diastolic arterial pressure (mmHg) < 60 mmHg (%)	80 ± 18	80 ± 18	76 ± 17	< 0.001
	14.6	14.4	17.5	0.102
Killip-Kimbal class ≥ 2 (%)	13.0	12.1	27.3	< 0.001
Creatinine (mg/dL) ≥ 2 mg/dL (%)	1 ± 0.8	1 ± 0.8	1.2 ± 0.9	< 0.001
	3.6	3.3	8.5	< 0.001
Hemoglobin (g/dL) ≥12 g/dL (%)	14.1 ± 1.8	14.1 ± 1.8	13.5 ± 2	< 0.001
	87.8	88.2	81.3	< 0.001

Values are mean ± SD, median (P25; P75) or n (%). AF: atrial fibrillation; AMI: acute myocardial infarction; LBBB: left bundle branch block.

**Figure 2:**
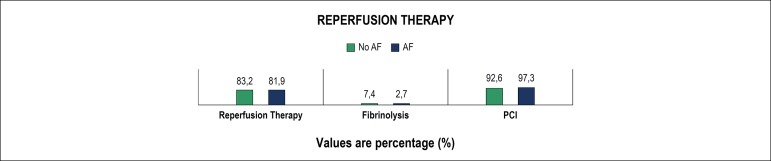
Reperfusion therapy. AF: atrial fibrillation; PCI: percutaneous coronary intervention.

Regarding therapy, reperfusion was successfully accomplished in both groups (Figure 2), with no significant difference. However, patients who developed AF had a longer time from symptom onset to reperfusion [median 243 (166-400) minutes vs. 267 (185-398) minutes, p = 0.033]. The number of patients who underwent coronary angiography or angioplasty was not statistically different. No difference in the number of affected vessels was found, although patients in group 2 had a higher prevalence of left main coronary artery disease (3.0% vs. 5.2%, p = 0.041), with no difference regarding other vessel disease. Patients in group 2 had implanted more bare-metal stents (25.1% vs. 35.4%, p = 0.003). Thrombectomy devices were more frequently used in patients in group 2 (32.5% vs. 42.9%, p < 0.001).

Medical therapy during in-hospital stay and at discharge are shown in Tables 3, 4 and 5. Most patients developed new-onset AF on the first day [median 0 (0-1) days; mean 1.2 ± 3.5 days].

**Table 3:** Medical therapy during in-hospital stay Values are percentage (%)

	All	Group 1 - No AF	Group 2 - New onset AF	p
Aspirin	98.6	98.6	99.2	0.349
Clopidogrel	86.7	86.6	89.3	0.138
Ticagrelor	17.6	17.9	12.3	0.042
Prasugrel	0	0	0	
Other AP	5.4	5.5	5.2	0.859
GPIIbIIIa inhibitors	33.6	33.5	35.8	0.362
UFH	46.3	46.3	45.6	0.784
Fondaparinux	6.1	6.0	7.7	0.185
Bivalirudin	0.7	0.6	0.8	0.512
LMWH	45.1	44.3	58.2	< 0.001
Vitamin K antagonists	1.3	1.0	5.2	< 0.001
NOAC	0.3	0.3	0.8	0.106
Beta-blockers	79.6	80.2	69.5	< 0.001
ACE inhibitors/ARB	86.3	86.6	80.8	0.002
Aldosterone antagonists	13.5	12.6	27.3	< 0.001
Statins	94.6	94.5	95.6	0.364
Diuretics	25.1	23.0	58.5	< 0.001
Amiodarone	6.5	2.9	65.9	< 0.001
Other antiarrhythmic drugs	1.0	0.9	1.9	0.08
Digitalis	0.9	0.3	11.3	< 0.001
Inotropes	7.8	6.6	27.3	< 0.001
Levosimendan	1.0	0.7	5.5	< 0.001

AF: atrial fibrillation; AP: antiplatelet therapy; GpIIb/IIIA inhibitors: Glycoprotein IIb/IIIa inhibitors; LMWH: low molecular weight heparin; UFH: unfractionated heparin.

**Table 4:** Medical therapy at discharge

	All	Group 1 - No AF	Group 2 - New onset AF	p
Aspirin	96.2	96.3	93.1	0.005
Clopidogrel	80.3	80.2	81.3	0.634
Ticagrelor	19.3	19.6	13.7	0.065
Prasugrel	0.3	0.3	0	1.000
Other AP	4.9	5.0	3.6	0.289
Vitamin K antagonists	2.4	1.9	11.2	< 0.001
NOACs	1.2	0.8	7.9	< 0.001
Beta-blockers	81.6	82.2	71.1	< 0.001
ACE inhibitors/ARB	87.7	88.0	81.3	< 0.001
Aldosterone antagonists	12.0	11.2	26.0	< 0.001
Diuretics	20.1	18.7	46.6	< 0.001
Amiodarone	2.4	1.1	26.4	< 0.001
Other antiarrhythmic drugs	0.1	0.1	0	1.000
Digitalis	0.4	0.2	4.0	< 0.001

Values are percentage (%). AF: atrial fibrillation; NOAC: non vitamin K anticoagulant.

**Table 5:** Patients from group 2 on triple therapy

Antithrombotic therapy	Group 2 - New onset AF
Aspirin + Clopidogrel + Vitamin K antagonists	10.2
Aspirin + Clopidogrel + NOACs	5.9
Aspirin + Ticagrelor/Prasugrel + Vitamin K antagonists	0
Aspirin + Ticagrelor/Prasugrel + NOACs	0
Total	16.1

Values are percentage (%). AF: atrial fibrillation; ACE inhibitors: angiotensin conversion enzyme inhibitors; ARB: angiotensin receptor blockers; NOAC: non vitamin K anticoagulant.

New-onset AF was associated with higher rate of in-hospital complications, namely: worse left ventricular function, higher prevalence of HF, cardiogenic shock, AV block, ventricular tachycardia, cardiac arrest, mechanical complications, stroke and major bleeding (Table 6).

**Table 6:** In-hospital complications

	All	Group 1 - No AF	Group 2 - New onset AF	p
LV Ejection fraction < 50%	43.4	42.2	62.1	p < 0.001
Heart failure	16.8	14.9	48.5	p < 0.001
Cardiogenic shock	5.9	4.9	22.3	p < 0.001
Atrioventricular block	5.3	4.8	14.2	p < 0.001
Ventricular tachycardia	2.6	2.1	10.7	p < 0.001
Cardiac arrest	5.2	4.8	11.8	p < 0.001
Mechanical complications	1.3	1.2	3.3	p = 0.002
Stroke	0.7	0.6	2.5	p = 0.001
Major bleeding	1.9	1.6	6.8	p < 0.001

Values are percentage (%). AF: atrial fibrillation; LV: left ventricular

**Figure 3:**
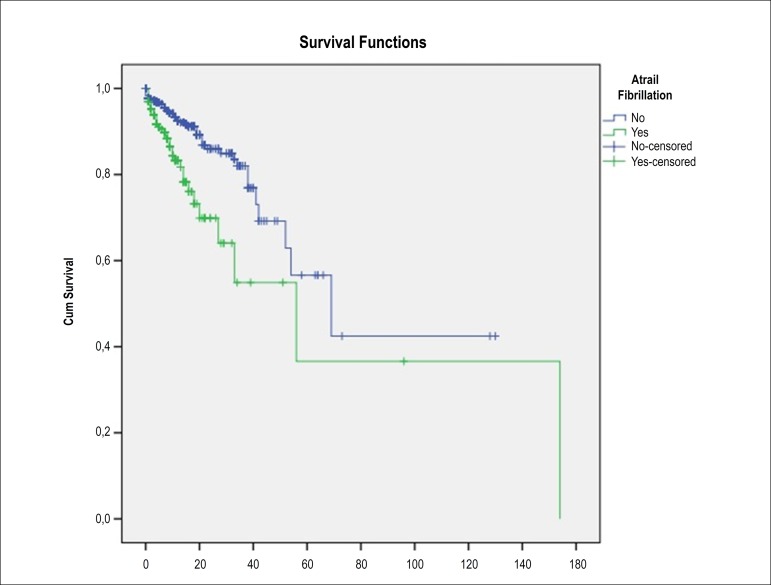
Kaplan-Meier survival curves for the two groups.

It was also associated to a more frequent need of Swan-Ganz catheter, intra-aortic balloon pump, invasive mechanical ventilation and transvenous cardiac pacing (Table 7). The duration of hospital stay was higher in patients of group 2 [3 (3; 5) days vs. 5 (4; 9) days, p < 0.001]. In-hospital mortality was higher in patients who developed new onset AF (3.8% vs. 13.4%; p < 0.001) (Figure 3). Although new-onset AF was associated with higher incidence of complications and in-hospital mortality, by multivariate analysis, new-onset AF was not found as an independent predictor of in-hospital mortality [OR 1.19 (0.62-2.27), p = 0.608].

**Table 7:** Invasive procedures performed

	OR (CI 95%)	p
Age	1.02 (1.01-1.04)	p < 0.001
Prior stroke	1.87 (1.09-3.21)	p = 0.023
Inferior located STEMI	1.57 (1.13-2.18)	p = 0.007
Complete AV block	1.94 (1.19-3.16)	p = 0.008

Values are percentage (%). AF: atrial fibrillation

We identified age, prior stroke, inferior located STEMI and complete AV block as independent predictors of new-onset AF (Table 8).

**Table 8:** Independent predictors of new onset atrial fibrillation

	All	Group 1 - No AF	Group 2 - New onset AF	p
Swan-Ganz catheter	0.6	0.4	3.6	p < 0.001
Intra-aortic balloon pump	1.2	1.1	2.7	p = 0.012
Invasive mechanical ventilation	3.5	3.2	9.0	p < 0.001
Transvenous cardiac pacing	3.6	3.1	11.2	p < 0.001

STEMI: ST-segment elevation myocardial infarction

## Discussion

These population-based data reflect the comprehensive experience of a community in a “real-life” setting and is more representative of those treated in clinical practice than populations enrolled in randomized controlled trials.

In our population, new-onset AF was found in 5.8% of patients hospitalized for STEMI, which is in line with recent published data.^1,7^ However, this number is lower than most older studies, suggesting that despite an increasingly older population with higher prevalence of comorbidities, the incidence of AF in the setting of acute MI has declined.^8,9^

New-onset AF was found more frequently in older patients, female sex, with higher prevalence of comorbidities, including hypertension, diabetes, previous stroke and previous heart failure and, therefore, higher CHA_2_DS_2_-VASc scores. According to Schmitt et al.,^1^ the use of interventional coronary revascularization has been associated with a notable decline in the AF incidence.^1^ However, one of the major findings of this study is that new-onset AF complicating STEMI is independent of reperfusion strategy. Reperfusion was successfully accomplished in both groups with no difference regarding the type of reperfusion and no difference in the number of affected or treated vessels between both groups. This is consistent with the OACIS study, which states that AF complicating the clinical course of MI appears to be irrespective of treatment by thrombolytics or PCI.^10,11^ However, there seems to be a favorable effect of early reperfusion therapy on preventing new-onset AF, as it also was suggested by a previous study.^8^ The use of thrombectomy devices, which may suggest a cardioembolic cause, were more frequently used in patients who developed AF, raising the question that some patients with new-onset AF may have had undiagnosed paroxysmal AF.^12^

There is insufficient data indicating preferences for rate or rhythm control in AF complicating STEMI. Besides beta-blockers, antiarrhythmic drug use is usually limited to amiodarone.^13^ In our population, two-thirds of the patients who developed new onset AF were started on amiodarone.

Selection of antithrombotic therapy was at the discretion of the attending physician and reflects a wide range of therapeutic strategies in a “real-life” setting. Considering the most recent guidelines published by the European Society of Cardiology, the recommendation is that STEMI patients with an indication for oral anticoagulation, as in AF, should be considered for triple therapy with aspirin, clopidogrel and oral anticoagulant for 1-6 months.^13,14^ As shown in Table 5, in this population, only 16.1% were on triple therapy at discharge, 10.2% with AAS, clopidogrel and vitamin K antagonists, and 5.9% on AAS, clopidogrel and non-vitamin K anticoagulant (NOAC). This low rate of triple therapy in STEMI patients with AF has been reported in other studies, such as the APEX-AMI study, where 10.6% of new-onset AF patients hospitalized for STEMI were on triple therapy.^15^ Seventeen percent of patients in group 2 were on P2Y_12_ inhibitors other than clopidogrel and none of these were on anticoagulants at discharge. It is noteworthy that 6.1% of patients on aspirin during in-hospital stay were not on aspirin when discharged, suggesting that a WOEST trial-like strategy was chosen, which is also supported by a meta-analysis that suggests that a combination of vitamin K antagonists and single antiplatelet therapy is the best choice for AF patients undergoing percutaneous coronary intervention (PCI) considering both efficacy and safety.^16,17^ Stenestrand et al.^18^ and Lopes et al.^15^ showed that only a small percentage of patients with AF and acute MI received oral anticoagulation therapy at discharge (30% and 43.4%, respectively). Our results show a lower rate of anticoagulation: only 20.6% of patients diagnosed with AF during in-hospital stay received oral anticoagulation at discharge. Forty-five percent of patients on anticoagulation at discharge were on NOACs.

Therapy with beta-blockers and angiotensin-conversion enzyme (ACE) inhibitors/angiotensin receptor blockers (ARBs) was less frequently found in patients who developed new-onset AF and although the higher incidence of complications may have limited therapeutic options, this could suggest that renin-angiotensin-aldosterone system inhibitors and beta-blockers may have a protective role, which is in line with previous studies.^1,19,20^ No difference was found regarding statin therapy, unlike what was suggested in previous studies.^21^

New-onset AF was associated with higher incidence of complications, namely stroke, heart failure, cardiogenic shock, arrhythmias, cardiac arrest, mechanical complications and major bleeding, and in-hospital mortality, but was not found as an independent predictor of mortality, suggesting that new-onset AF complicating STEMI may simply be an indicator of poor overall clinical status, which is consistent with previous studies.^5,22^ Studies on critically ill patients also suggest new-onset AF is an indicator of clinical severity and poorer prognosis.^4,23-25^

Age, prior stroke, inferior STEMI and complete AV block were found as independent predictors of new-onset AF.

Age and prior stroke reflect patients with more comorbidities. A systematic review by Schmitt et al.^1^ of 20 publications also found that new-onset AF is more likely to complicate AMI in older patients.^1^ Anatomic atrial abnormalities with aging, such as atrial fibrosis and smooth muscle cell proliferation, may provide anatomic substrate for the multiple wavelet re-entry mechanism of AF.^26^

Logistic regression analysis showed that inferior STEMI was an independent predictor of new-onset AF in our population. Kyriakidis et al.^27^ found that all patients that developed supraventricular arrhythmia had inferior STEMI and right and left atrial ischemia, and concluded that ischemia of the sinus node due to coronary occlusion proximal to the origin of the sinus node artery was probably one of the underlying causes of atrial fibrillation.^27^ According to Tjandrawidjaja et al.,^28^ the compromised anatomy of the principal coronary atrial branches is associated with the development of atrial arrhythmia.^28^

The GUSTO III study found that the development of AF could be due to other post-AMI complications occurring before AF, such as complete AV block.^26^ An association between complete AV block and incidence of AF has also been reported previously, particularly in AH type of AV block, due to lesions observed in the atrial muscles including the internodal tracts.^29^

The main question of long-term management of new-onset AF in STEMI and whether to start anticoagulation therapy in these patients remains unanswered, as there is not enough data. In our population, only one in five patients was discharged on oral anticoagulation. Follow-up data on these patients is needed to assess long-term complications. According to some studies, new-onset AF in the acute setting may indicate a propensity to develop arrhythmia again, potentially because of associated comorbidities or other predisposing factors.^3^ In our population, the therapeutic strategy seems to have been based on the notion that new-onset AF in STEMI could represent a transient complication of acute coronary syndrome, as suggested by some studies that have shown that in patients with coronary artery disease, atrial ischemia independently promotes the formation of a substrate for AF and that atrial impulse conduction abnormalities were abolished after reperfusion was achieved with either thrombolysis or primary percutaneous coronary intervention (PCI).^8,30,31^

### Study limitations

Although data have been prospectively acquired, the main limitations of this study derive from its retrospective design, which limits some available data.

Another limitation is that some patients diagnosed with new-onset AF may in fact have previous yet undiagnosed paroxysmal AF, which may lead to an overestimated incidence. These patients may also represent a different subtype of patients, compared to actual new-onset AF due to the ischemic event.

## Conclusions

This study reflects a very large registry of new-onset AF in patients hospitalized for STEMI. It shows that in a modern revascularization era, AF is still a frequent complication of acute myocardial infarction. It can be reduced by early reperfusion independently of reperfusion strategy. These registry data confirm the high mortality and morbidity associated with AF, which may suggest the importance of identifying this subgroup of STEMI patients. Age, prior stroke, inferior STEMI and complete AV block were found as independent predictors of new-onset AF in the setting of STEMI. Furthermore, data indicates that only a minority of patients receive oral anticoagulants at discharge. Although the incidence has decreased, we can still expect AF to remain a frequent and worrisome complication of AMI. Further studies answering questions such as how to reduce the risk of AF development, whether the use of new generation drug-eluting stents and modern antithrombotic strategies aimed at better reperfusion will reduce the incidence of new-onset AF during AMI and how to determine the optimal use of oral anticoagulation therapy in combination with antiplatelet therapy in this setting.
